# Discovery of lung adenocarcinoma tumor antigens and ferroptosis subtypes for developing mRNA vaccines

**DOI:** 10.1038/s41598-024-53622-y

**Published:** 2024-02-08

**Authors:** Yan Chen, Changwen Zhang, Yu Li, Xiaoyu Tan, Wentao Li, Sen Tan, Guangnan Liu

**Affiliations:** Department of Pulmonary and Critical Care Medicine, The Second Affiliated Hospital of Guangxi Medical University, Daxue East Road No.166, Nanning, 530007 Guangxi China

**Keywords:** Cancer, Immunology

## Abstract

mRNA vaccines are becoming a feasible alternative for treating cancer. To develop mRNA vaccines against LUAD, potential antigens were identified and LUAD ferroptosis subtypes distinguished for selecting appropriate patients. The genome expression omnibus, cancer genome atlas (TCGA) and FerrDB were used to collect gene expression profiles, clinical information, and the genes involved in ferroptosis, respectively. cBioPortal was used to visualize and compare genetic alterations, GEPIA2 to calculate prognostic factors of the selected antigens, and TIMER to visualize the relationship between potential antigens and tumor immune cell infiltration. Consensus clustering analysis was utilized to identify ferroptosis subtypes and their prognostic value assessed by Log-rank and cox regression tests. The modules of ferroptosis-related gene screening were conducted by weight gene co-expression network analysis. The LUAD ferroptosis landscape was visualized through dimensionality reduction and graph learning. Six tumor antigens had obvious LUAD-mutations, positively correlated with different antigen-presenting cells, and might induce tumor cell ferroptosis. LUAD patients were stratified into three ferroptosis subtypes (FS1, FS2, and FS3) according to diverse molecular, cellular, and clinical characteristics. FS3 showed the highest tumor mutation burden and the most somatic mutations, deemed potential indicators of mRNA vaccine effectiveness. Moreover, different ferroptosis subtypes expressed distinct immune checkpoints and immunogenic cell death modulators. AGPS, NRAS, MTDH, PANX1, NOX4, and PPARD are potentially suitable for mRNA vaccinations against LUAD, specifically in patients with FS3 tumors. This study defines vaccination candidates and establishes a theoretical basis for LUAD mRNA vaccinations.

## Introduction

Lung cancer is one of the most malignant tumors with the highest morbidity and mortality worldwide^1^. Systematic treatment of lung cancer has been difficult to develop due to the heterogeneity of the disease, patient complications, and drug safety, tolerability, and efficacy^2^. Lung cancer patients tend to be older on average than those with breast or colon cancer, and are mostly smokers with heart disease and emphysema, leading to increased all-cause mortality and asthenia after surgery^3^. The combination of these factors reduces the patient’s tolerance to medical or surgical treatment. With the effective application of targeted therapy and immunotherapy, immune checkpoint inhibitors (ICIs) have demonstrated significant advantages in non-small cell lung cancer (NSCLC) therapy. However, it is difficult to predict whether a patient will respond to ICIs.

Lung adenocarcinoma (LUAD), the most common subtype of lung cancer, has a 5-year survival rate of less than 20% worldwide. Finding a new treatment method for LUAD is crucial because current therapies such as chemotherapy, radiation, targeted therapy, and immunotherapy are gradually leading to therapy resistance. Currently, it is widely believed that ferroptosis, a type of iron-dependent programmed necrosis, plays an important part in the occurrence and progression of many malignant tumors^4^. The interaction amongst cancer cells, microenvironment, and host immune status is complicated in ferroptosis. Therefore, efforts are needed to collaboratively create multidimensional immune landscapes associated with ferroptosis and predict biomarkers for individualized immunotherapy.

mRNA vaccines have emerged as an effective cancer immunotherapy method. Due to its high efficacy, safe administration, quick development potential, and economical manufacture, the mRNA cancer vaccine outperforms other traditional vaccination platforms^5^; however, its application in LUAD remains largely unclear. Many studies indicate that immunotyping may reveal the immunological state and immune microenvironment of tumors, strongly related to vaccination potential and therapeutic response. To date, no mRNA vaccine has been formulated against ferroptosis-related antigens in LUAD, and no patient subpopulation fit for immunization has been discovered.

Here, LUAD ferroptosis subtypes were determined to select appropriate patients from a heterogeneous population and to discover possible LUAD antigens to create an mRNA vaccine. Among the LUAD mutant and overexpressed genes, we isolated six candidates related to antigen presenting cell (APC) infiltration and poor survival. We subsequently identified three LUAD ferroptosis subtypes, with different clinical, molecular, and cellular features, and confirmed them in an independent cohort based on the clustering of genes associated with ferroptosis. Finally, the LUAD ferroptosis landscape was established by evaluating the distribution of related genetic traits among individual patients. Our study could precisely determine the ferroptosis status of each LUAD patient and estimate their prognosis, which is beneficial when selecting mRNA vaccine personalized treatment.

## Methods

### Data extraction and preprocessing

The “FPKM” data type was selected when downloading the cancer genome atlas (TCGA) data sets of 585 LUAD patient from UCSC Xena (https://xenabrowser.net/datapages/). Genomic Data Commons (GDC) data sets were used to extract “Primary solid tumor” (01A) information and convert it to a “Transcripts Per Million” (TPM) format. The somatic mutation data for LUAD patients were chosen as "Masked Somatic Mutation" data and pretreated by using the VarScan program (version 2.4.0), while the somatic mutation was displayed using the maftools R package (maftools 2.14.0). Age, TNM-staging, survival status and longevity information was downloaded along with the patient's clinical data. After excluding patients without clinical information, 513 patients with survival information and 507 patients with other clinical information were retained. The gene expression data of GSE11969^6^ and patient clinical information, such as survival time and survival status, were obtained from the gene expression omnibus (GEO) database. Samples from humans were chosen, and the chip platform was based on GPL7015. Subsequently, 163 tumor samples were retained and included, and the microarray data were standardized using the limma^7^ package of R (limma 3.54.2).

**Figure 1:**
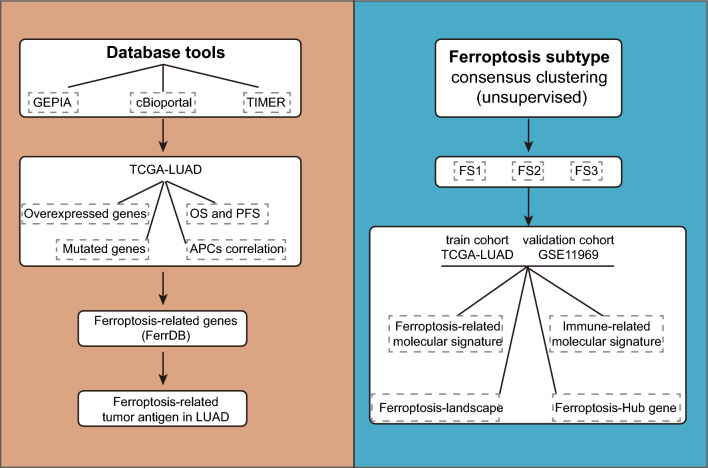
Flow chart.

Finally, 424 ferroptosis-related genes (FRGs) including driver genes, suppressor genes, and markers were extracted from FerrDB (www.zhounan.org/ferrdb/)^8^. Genes for immune checkpoint (ICP) and immune cell death (ICD) were acquired from previous studies^9,10^. The design of the study is depicted in Fig. 1.

### GEPIA and cBioPortal analysis

To prevent data imbalance and ineffective differential analysis, original RNA-Seq data from the TCGA database were recalculated using UCSC Xena (July 8, 2019). Differential gene expression was integrated using Gene Expression Profiling Interactive Analysis (GEPIA, http://gepia2.cancer-pku.cn)^11^. The value of differentially expressed genes between |log2FC|> 1 and *p* values 0.01 was determined using an analysis of variance (ANOVA). Overall survival (OS) and progression-free survival (PFS) were assessed by using the Kaplan–Meier algorithm with a 50% cut-off for low- and high-expression groups. Comparative analysis was performed using the Log-rank test, and statistical significance was set at *p* < 0.05.

The RNA-seq raw data from the TCGA was integrated with the cBio Cancer Genomics Portal (http://www.cbioportal.org)^12^ to analyze genetic alterations in LUAD, from which the microsatellite instability and TMB data of TCGA-LUAD patients were extracted (statistical significance was set at *p* < 0.05).

### Timer analysis

The comprehensive resource Tumor Immune Estimation Resource (TIMER, https://cistrome.shinyapps.io/timer/)^13^ allows for systematic analysis of immunological infiltrates in a variety of cancers. Here, TIMER was applied to analyze and depict the relationship between the quantity of tumor-infiltrating immune cells (TIICs) and potential tumor antigens by using the analytic modules of gene expression, somatic mutations, somatic copy number alterations and clinical outcomes. The purity adjustment was conducted using the partial Spearman's correlation, and statistical significance was set at *p* < 0.05.

### Identification and verification of ferroptosis subtypes

The “ConsensusClusterPlus” R package^14^ was used to cluster the genes associated with ferroptosis based on the expression profile, and a consensus matrix was created to determine the corresponding ferroptosis subtypes and gene modules. 80% of patients in the cohort participated in each of 1000 bootstraps by utilizing the partition around the medoids algorithm and the “1-Pearson correlation” distance measure. The best partition was revealed by analyzing the consensus matrix and the consensus cumulative distribution function, with cluster sets ranging from 2 to 10. The ferroptosis subtypes were subsequently confirmed in a separate GEO cohort under identical conditions.

### Prognostic evaluation of ferroptosis subtypes

A Log-rank test and univariate and multivariate Cox regression were utilized to assess the prognostic values of the ferroptosis subtypes, with stage and grade as covariates and OS and PFS as the endpoints. An ANOVA was applied to evaluate the correlation between ferroptosis subtypes and distinct immune-related molecular and cellular signatures. Immune enrichment scores were determined for every sample using single-sample Gene Set Enrichment Analysis GSEA (ssGSEA) from the Gene Set Variation Analysis (GSVA) software (version1.46.0)^15^.

### Gene co-expression network

Modules of genes linked to ferroptosis were screened using the "weighted correlation network analysis (WGCNA)" R program (Version 1.70-3)^16^. The pickSoftTreshold function was utilized to choose the soft threshold for building the scale-free network, and the best value was six. A topological matrix was then created, and hierarchical clustering was used to generate Eigengenes. According to module Eigengenes, the correlation between modules was built, and hierarchical clustering was completed. An analysis of functional enrichment was done through the Metascape database (www.metascape.org/)^17^, which includes Gene Ontology (GO) and the Kyoto Encyclopedia of Genes and Genomes (KEGG)^18,19^. Adjusted *p* < 0.05 was regarded as statistically significant for enrichment.

### Construction of ferroptosis landscapes in tumor microenvironments

The distribution of ferroptosis subtypes in LUAD patients was visualized through a dimensionality reduction analysis based on graph learning, and a dimensionality reduction function based on the Monocle package^20^ with Gaussian distribution. The discriminant dimensionality reduction method known as “DDRtree” was applied, and the optimum number of components was set at four. Finally, a functional map of cell trajectories of different ferroptosis subtypes was colored to visualize the ferroptosis landscape.

### Statistical analyses

R software 4.4.1 was utilized to conduct all statistical analyses, and the Wilcoxon rank-sum test was applied to compare group differences. Differences among more than two groups were assessed by the Kruskal–Wallis test, and correlation was performed by Spearman’s correlation method (statistical significance was set at *p* < 0.05).

### Ethics approval and consent to participate

This study does not contain any studies with human participants or animals performed by any of the authors.

## Results

### Discovery of potential ferroptosis-related LUAD tumor antigens

**Figure 2:**
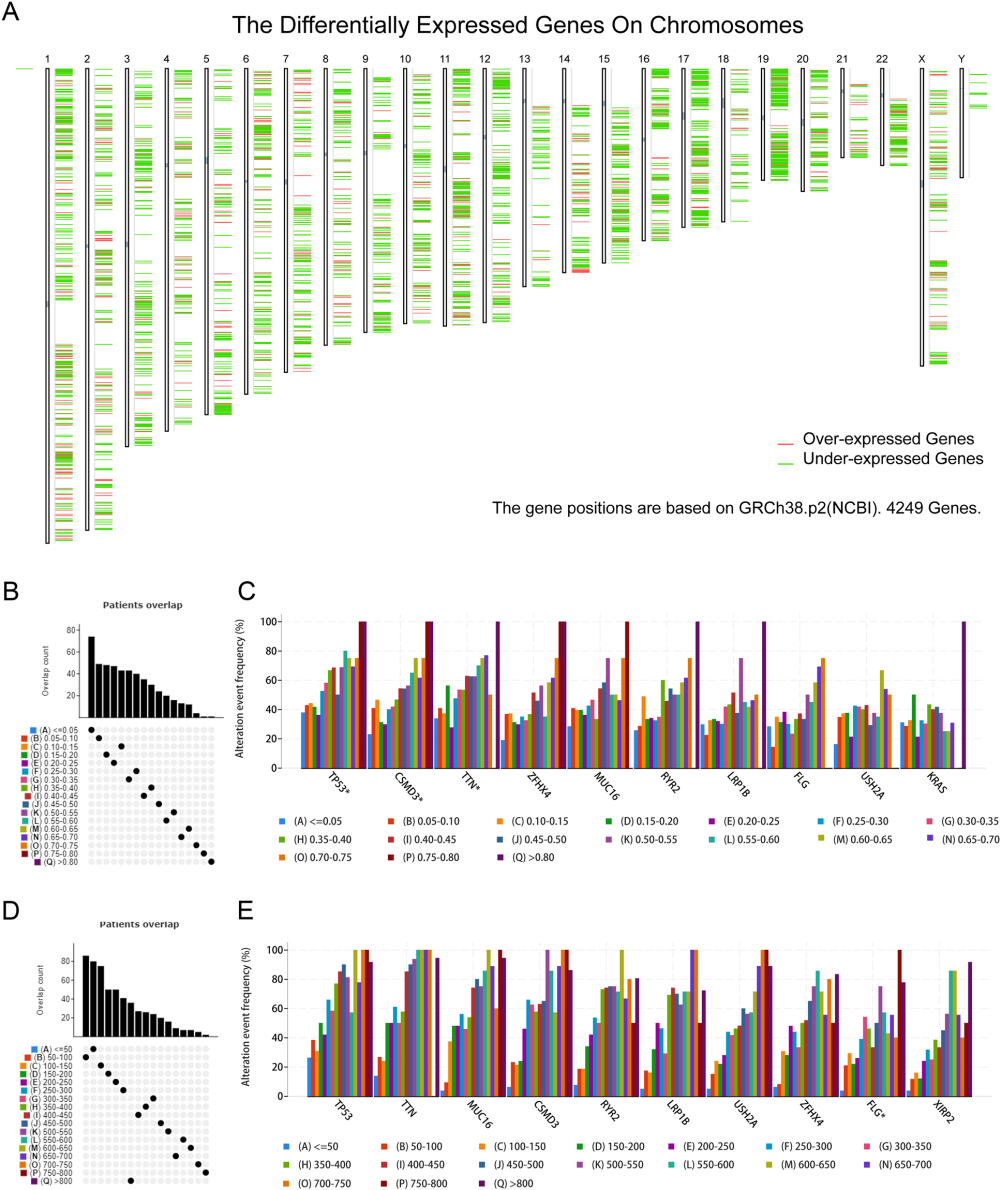
Identification of potential LUAD tumor antigens. (**A**) tumor-associated antigens (chromosomal distribution of up- and down-regulated genes in LUAD as indicated). (**B**–**E**) tumor-specific antigens (samples overlapping in (**B**) altered genome fraction and (**D**) mutation count groups). Genes with the highest frequency in (**C**) altered genome fraction and (**E**) mutation count groups.

To discover a viable LUAD mRNA vaccine, we screened for excessively expressed genes. Among the 4469 differentially expressed genes, 1109 genes most likely to encode tumor-associated antigens were found to be potentially overexpressed (Fig. 2A). An analysis of mutant gene segments and mutation rates in individual samples was then used to filter out a total of 14733 altered genes that could be responsible for producing tumor-specific antigens (Fig. 2B and D). The tumor protein p53 (TP53) was the most often mutated gene according to mutational analyses, both in regards to the mutant genome proportion and mutation counts (Fig. 2C and E). CSMD3, TTN, ZFHX4, MUC16 and LRP1B were also significantly mutated in LUAD patients, both in number and frequency. Considering the long full length of the TTN and MUC16 genes, the specificity is low, while other gene mutations might have important significance in LUAD. Overall, a total of 734 cancer-related genes were identified to be up-regulated and frequently mutated, which might serve as potential tumor antigens.

**Figure 3:**
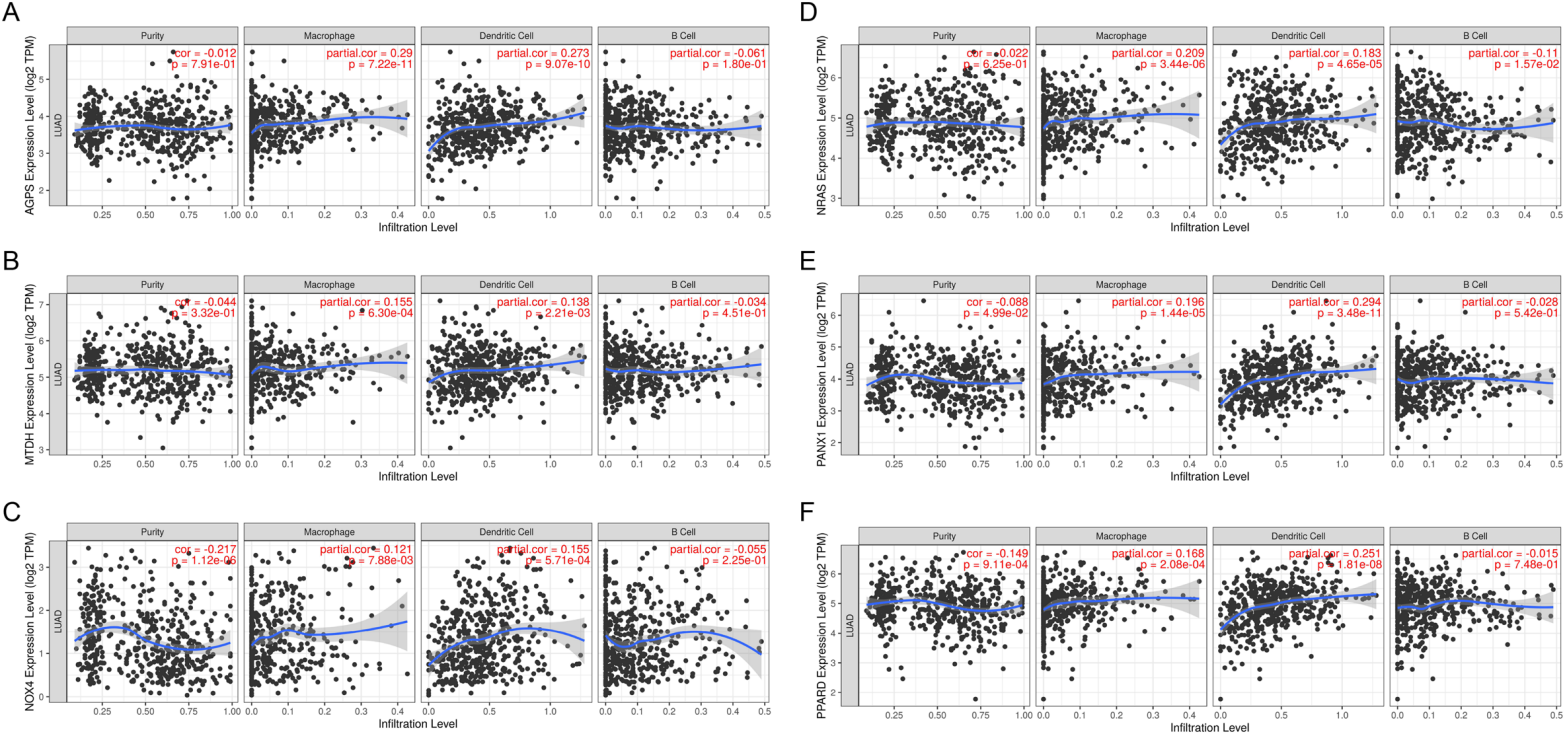
Identification of tumor antigens associated with APCs. Correlation between the expression levels of (**A**) AGPS, (**B**) MTDH, (**C**) NOX4, (**D**) NRAS, (**E**) PANX1, and (**F**) PPARD and infiltration of macrophages, dendritic cells, and B cells in LUAD tumors.

**Figure 4:**
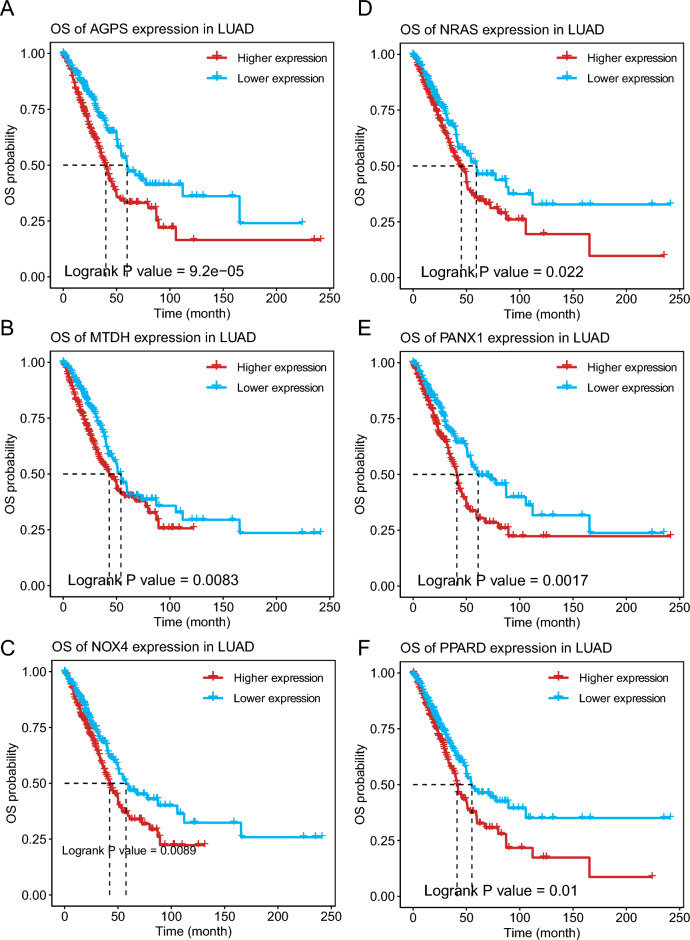
Identification of tumor antigens associated with LUAD prognosis. (**A**–**F**) Kaplan–Meier curves showing OS of LUAD patients stratified on the basis of (**A**) AGPS, (**B**) MTDH, (**C**) NOX4, (**D**) NRAS, (**E**) PANX1, and (**F**) PPARD expression levels.

**Figure 5:**
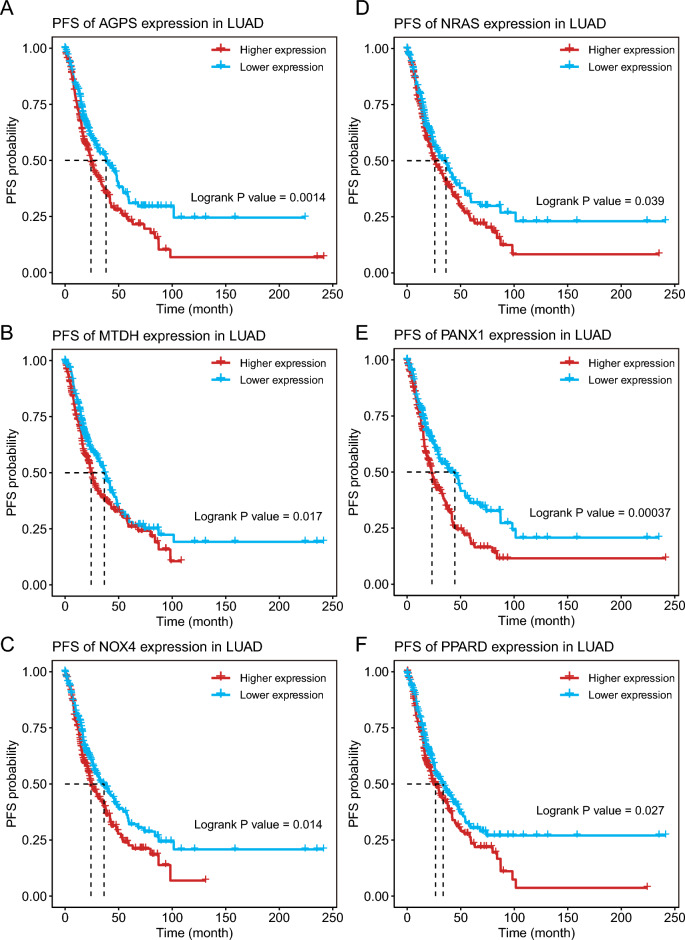
Identification of tumor antigens associated with LUAD prognosis. (**A**–**F**) Kaplan–Meier curves showing PFS of LUAD patients stratified on the basis of (**A**) AGPS, (**B**) MTDH, (**C**) NOX4, (**D**) NRAS, (**E**) PANX1, and (**F**) PPARD expression levels.

**Figure 6:**
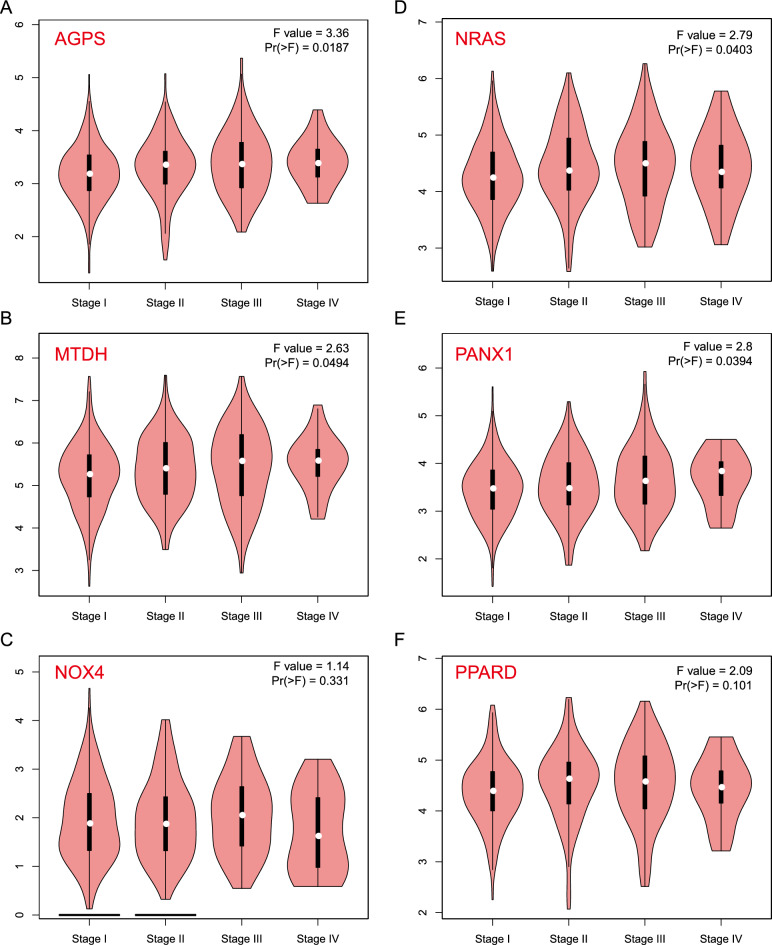
Relationship between potential tumor antigens and tumor TNM staging. The expression of (**A**) AGPS, (**B**) MTDH, (**C**) NOX4, (**D**) NRAS, (**E**) PANX1, and (**F**) PPARD in different stages was based on the GEPIA database.

The possibility of the ferroptosis gene as an mRNA antigen was then investigated. After survival analysis of OS and PFS, 24 FRGs were identified from 734 potential antigens. Considering the interaction between tumor antigens and antigen presenting cells (APCs), these potential antigens were further screened for APC association through the TIMER database where it was found that the AGPS, NRAS, MTDH, PANX1, NOX4, and PPARD genes showed a positive correlation with different APCs (Fig. 3A–F), and the prognosis was poorer for the genes with high expression (Figs. 4A–F and 5A–F). Furthermore, the expression of these genes showed different trends at different TNM stages and increased expression trends at advanced stages (Fig. 6A–F). These results indicate that the recognized tumor antigens might be processed and presented to T cells directly by APCs and then recognized by B cells to trigger an immune response. Ultimately, these genes might induce ferroptosis in tumor cells to kill them.

### Identification of potential LUAD ferroptosis subtypes

**Figure 7:**
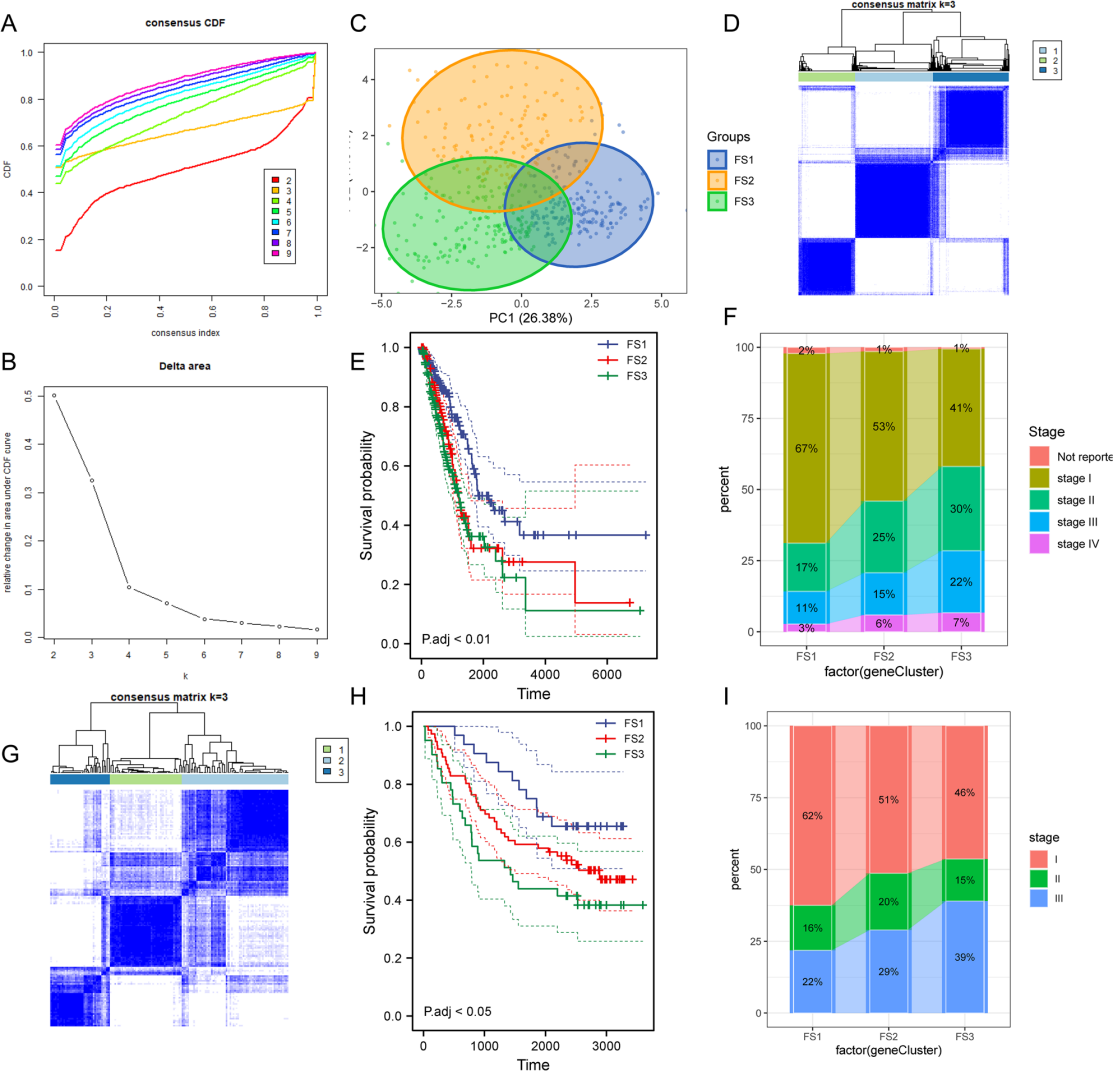
Identification of potential LUAD ferroptosis subtypes. (**A**) Cumulative distribution function curve. (**B**) δ-area of FRGs in the TCGA cohort. (**C**) 2D-PCA plot of the sample distribution. (**D**) Sample clustering heat map. (**E**) Kaplan–Meier curves showing OS of LUAD ferroptosis subtypes in the TCGA cohort. (**F**) Distribution of FS1–FS3 in LUAD stages. (**G**) Consistent clustering heat map for K = 3 in the GEO cohort. (**H**) Kaplan–Meier curves showing OS of LUAD ferroptosis subtypes in the GSE11969 cohort. (**I**) Distribution ratio of FS1–FS3 in LUAD stages.

Ferroptosis has recently been proved to be crucial in tumor killing and is strongly connected to tumor immunity. Ferroptosis subtypes could reflect the ferroptosis state of malignancies and their microenvironment, assisting in the identification of patients who are vaccine candidates. Therefore, consensus clusters were constructed by analyzing the expression profiles of 424 ferroptosis-associated genes in TCGA-LUAD. We chose k = 3 based on their cumulative distribution function and functional triangle area, where immune-related genes stably clustered (Fig. 7A,B), resulting in three ferroptosis subtypes, identified as FS1, FS2, and FS3 (Fig. 7C,D). FS1 had a better prognosis, whereas FS2 and FS3 had a lower probability of survival (Fig. 7E). The distribution of subtypes according to tumor stage and grade showed an irregular clustering of patients with stage diagnoses, while FS2 and FS3 subtypes accounted for a higher percentage of patients in the middle and late stages (Fig. 7F). In accordance with the findings of the TCGA-LUAD cohort, GSE11969 could be divided into FS1, FS2, and FS3 ferroptosis subtypes (Fig. 7G), which also had prognostic relevance (Fig. 7H) and significant alteration at different stages (Fig. 7I). In summary, ferroptosis classification could be utilized to estimate LUAD patient prognosis with better accuracy than traditional classification and staging, and this has been validated in different cohorts.

### Association of ferroptosis subtypes with tumor mutation

**Figure 8:**
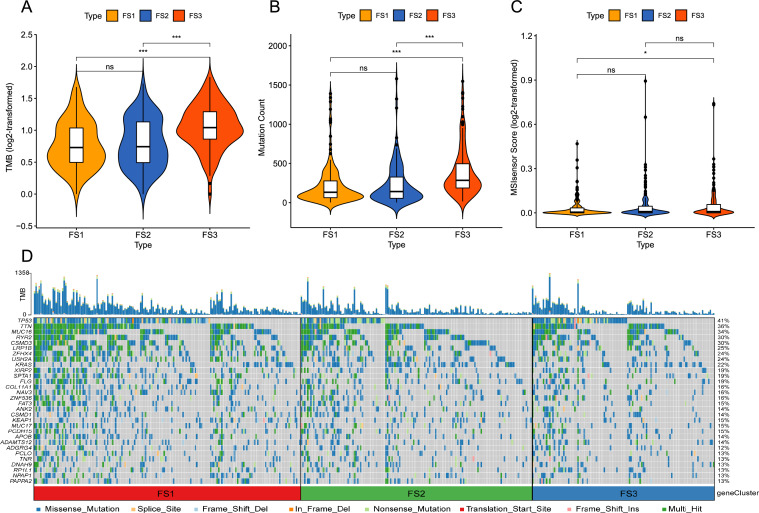
Association of ferroptosis subtypes with TMB and mutations. (**A–C**) Comparison of TMB (**A**), mutation count (**B**), and MSI (**C**) in different ferroptosis subtypes in LUAD. (**D**) LUAD waterfall plot showing the mutation signature genes of different ferroptosis subtypes (FS1, FS2 and FS3). *p < 0.05, **p < 0.01, ***p < 0.001.

Stronger anti-cancer immunity is correlated with higher TMB and somatic mutation rates. Thus, we calculated the TMB, MSI, and total number of mutations for each LUAD patient by utilizing the TCGA-LUAD mutation dataset and comparing three ferroptosis subtypes. In Fig. 8A and B, FS3 had the highest TMB and total number of mutations, followed by FS2 with FS1 having the lowest, and there were significant statistical differences among the three. There was no significant difference in MSI among the three subtypes, and FS3 patients had a higher MSI than FS1 patients (Fig. 8C). Additionally, 30 genes, including TP53, TTN, MUC16, etc., also showed different mutation status in different subtypes (Fig. 8D). These results suggest that the TMB and number of mutations might be used as possible markers for the mRNA vaccination and that different ferroptosis subtypes have different mutation characteristics.

### Correlation of ferroptosis subtypes with ICPs and immunogenic cell death modulators

**Figure 9:**
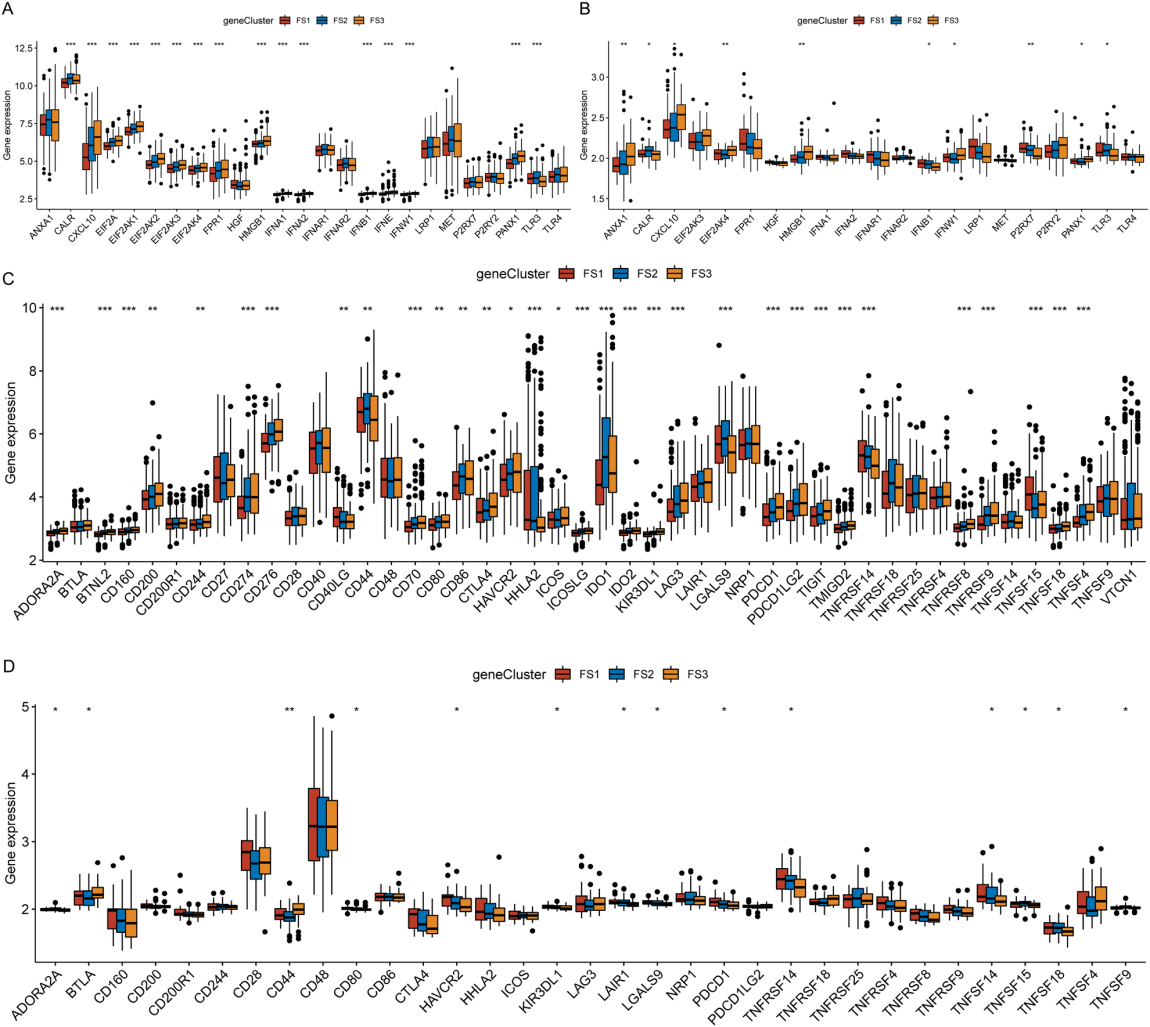
Association of ferroptosis subtypes with ICD and ICP regulatory genes. (**A**,**B**) Differences in ICD modulator expression between the three ferroptosis subtypes in the TCGA-LUAD (**A**) and GSE11969 cohorts (**B**). (**C**,**D**) Differences in ICP expression of three ferroptosis subtypes in the TCGA-LUAD (**C**) and GSE11969 cohorts (**D**). *p < 0.05, **p < 0.01, ***p < 0.001, ****p < 0.0001.

Recent research has demonstrated that immunological checkpoints (ICPs) and immunogenic cell death (ICD) modulators play a significant role in modulating anti-tumor immunity in the host and influencing the efficacy of mRNA vaccines. Due to the important relationship between ferroptosis and immune regulation, the different expression of ICP and ICD modulators was evaluated in the 3 ferroptosis subtypes. In the TCGA-LUAD and GSE11969 cohorts, 25 (Fig. 9A) and 21 (Fig. 9B) ICD genes were identified, respectively, and we observed almost the same expression trend of differentially expressed ICD genes in different subtypes; they were most expressed in FS3, followed by FS2 and then FS1. Among these, CXCL10 and HMGB1 showed significant differences between the two cohorts. Additionally, 46 (Fig. 9C) and 33 (Fig. 9D) ICP genes were found in the TCGA-LUAD and GSE11969 cohorts, respectively, and more differentially expressed ICP genes were observed in the TCGA-LUAD cohort, which might be related to the larger sample size. As shown in Fig. 9C and D, ICP gene expression trends were similar in the two cohorts; however, different trends were observed in the different ferroptosis subtypes.

### Cellular and molecular features of the ferroptosis subtypes

**Figure 10:**
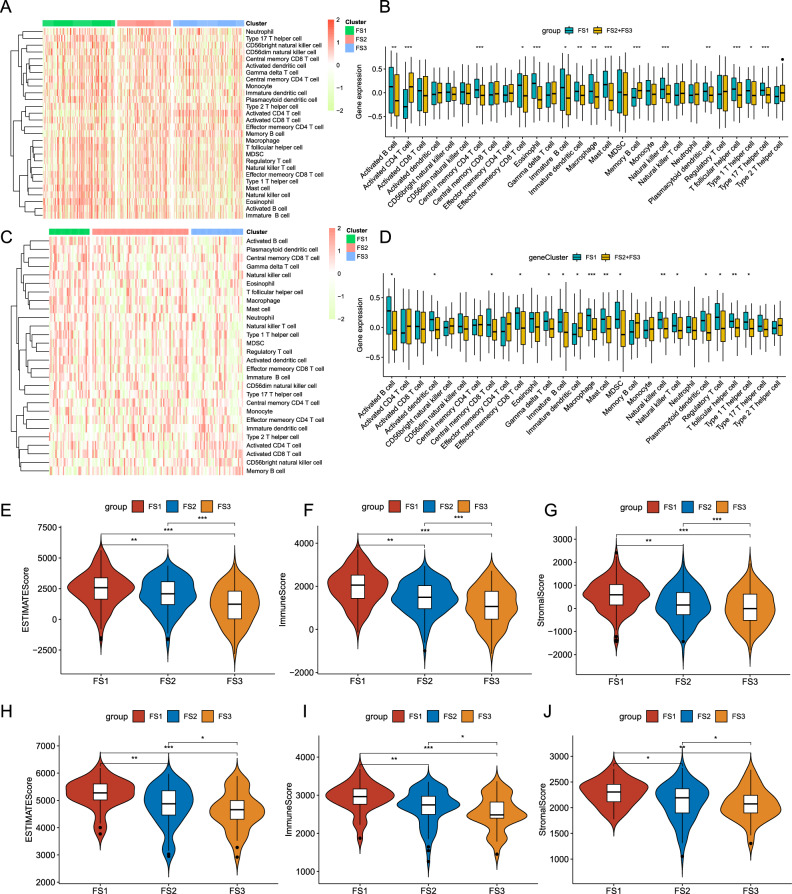
Cellular and molecular characteristics of ferroptosis subtypes. (**A** and **C**) Heatmaps of enrichment scores for 28 immune cell markers of LUAD immune subtypes in (**A**) TCGA-LUAD and (**C**) GSE11969 cohorts. (**B** and **D**) Differential enrichment scores for 28 immune-related cell markers in (**B**) TCGA-LUAD and (**D**) GSE11969 cohorts. (**E**–**G**) ESTIMATE, Immune, and Stromal scores in the TCGA-LUAD cohort. (**H**,**J**) ESTIMATE, Immune, and Stromal scores in the GSE11969 cohort. * p < 0.05, **p < 0.01, ***p < 0.001.

The LUAD immunological activity affects how the body reacts to mRNA vaccinations; therefore, ssGSEA was used to score 28 previously reported signature genes in the TCGA-LUAD and GSE11969 cohorts to further describe the immune cell components in the three ferroptosis subtypes. The immune cells were separated into three groups, as seen in Fig. 10A,B. In the TCGA-LUAD cohort, FS2 and FS3 displayed comparable immune cell ratings. The immune cell composition differed significantly amongst the subtypes. The scores of activated B cells, macrophages, mast cells, and natural killer cells in FS1 were significantly higher than those in FS2 and FS3 (*p* < 0.01), while the scores of activated CD4 T cells and memory B cells in FS3 were higher than those in FS1. A similar trend of immune cell infiltration was observed in the GSE11969 cohort (Fig. 10C,D). Based on the ESTIMATE algorithm, we found that patients with FS1 tumors had more immune cell infiltration, followed by FS2 and then FS3 (Fig. 10E,F). Additionally, the infiltration of stromal cells was highest in FS1, followed by FS2 and then FS3 (Fig. 10G). Thus, FS1 is a "hot" immune phenotype, whereas FS3 is a "cold" immune phenotype, and FS2 is in between them. The same differential trend was shown in the GSE11969 cohort (Fig. 10H–J). These findings imply that ferroptosis subtypes reflect the LUAD immunological state and could help select patients who would benefit from mRNA immunization. Immune infiltration could be induced by mRNA vaccines containing these antigens in patients with cold FS3 type malignancies.

### The LUAD ferroptosis landscape

**Figure 11:**
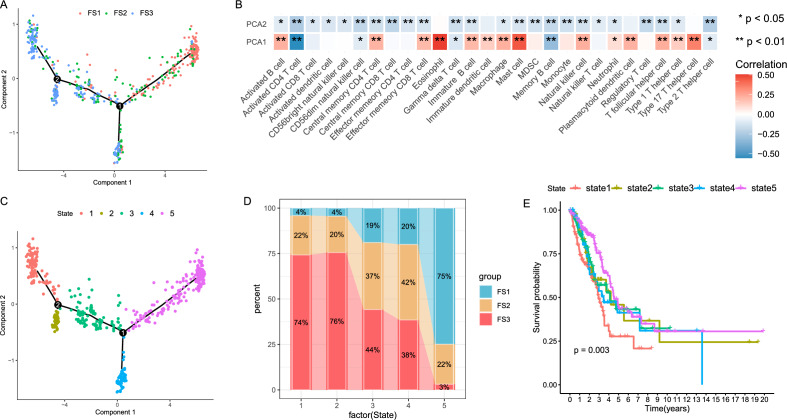
The ferroptosis landscape of LUAD. (**A**) The position of each patient in the immune profile, with colors corresponding to the ferroptosis subtypes identified above, represents the overall characteristics of Ferroptosis-related microenvironment (FRME). (**B**) Association of PCA1/2 with immune modules. **C** Patients with LUAD, reclassified according to their location, were divided into states 1, 2, 3, 4, and 5. (**D**) Proportion of patients by ferroptosis classification according to state. **E** Patient prognostic curves in different states (the patient prognosis differences in different states could be seen).

The ferroptosis gene expression profile of each patient was used to construct a ferroptosis profile for LUAD (Fig. 11A). As illustrated in Fig. 11B, the horizontal axis (PCA1) was associated with several immunological cells, among which activated B cells, eosinophils, macrophages, mast cells and follicular T cells were the most positively correlated, while the vertical axis was negatively correlated with most immune cell infiltration. Within the same subtype, the presence of different intra-cluster heterogeneity was also observed. According to the trajectory position of the sample group, all the samples were further divided into five states (Fig. 11C), and their proportions in different ferroptosis subtypes are shown in Fig. 11D. Prognostic analysis revealed that the survival curves of the five states differed significantly in terms of survival (*p* < 0.01), with state 1 having the worst prognosis (Fig. 11E). Collectively, the ferroptosis profile based on ferroptosis subtypes could effectively analyze the ferroptosis status of each LUAD patient and estimate their prognosis, facilitating the personalized selection of mRNA vaccine.

### Identification of LUAD ferroptosis gene co-expression modules and ferroptosis hub genes

**Figure 12:**
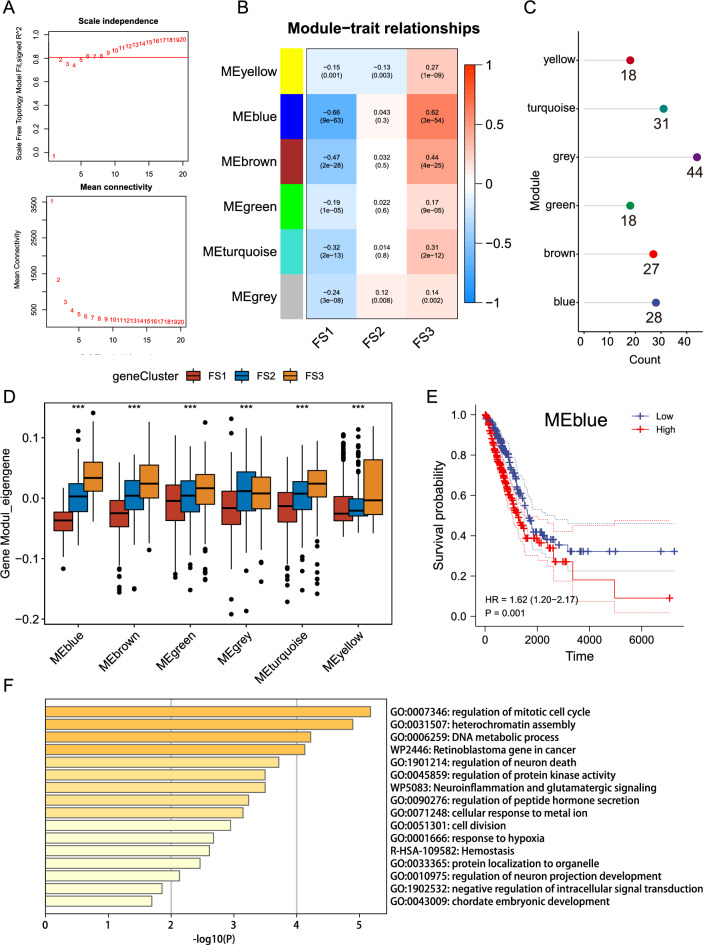
Identification of ferroptosis hub genes of LUAD. (**A**) The optimal soft threshold value of WGCNA was determined. (**B**) The six modules obtained by WGCNA. (**C**) The number of genes in the six modules. (**D**) Comparison of scores of ferroptosis subtypes in different modules. (**E**) The prognostic curve of the MEblue module showed a significant difference. (**F**) Functional enrichment of genes in the MEblue module.

Identification of key FRGs could help oncologists determine whether patients are candidates for the mRNA vaccination. We built a WGCNA of FRGs to find the important genes. The soft threshold was set to six in the scale-free network (Fig. 12A). The gene matrix is transformed into the adjacency matrix and the adjacency matrix into the topological matrix. At least 10 genes were set for each gene module. Gene characteristics were calculated for each module, and similar modules were merged. Finally, six modules in total were obtained, of which the grey module was the unassigned gene (Fig. 12B,C). When the Eigengene scores of modules were compared, FS3 had the highest score, except for the gray module, followed by FS2 and FS1 (Fig. 12D). A subsequent prognostic analysis found that only the blue module had significant differences between its score and prognosis (Fig. 12E). The genes in the blue module were significantly enriched in ferroptosis-related functions and pathways, among which cell-cycle regulation (GO:0007346, GO:0031507, GO:0006259) and protein-kinase regulation were related to tumors (Fig. 12F). The genes in the blue module include PAQR3, CISD2, IFNG, DNAJB6, RRM2, MUC1, GLRX5, TFAP2A, HELLS, HILPDA, NRAS, COPZ1, EZH2, FANCD2, AHCY, HSF1, CFL1, SLC16A1, TIMM9, TFR2, SRSF9, KIF20A, CDC25A, SUV39H1, SLC38A1, MMD, CDCA3, and PARP2. Therefore, hub genes could be exploited as biomarkers to determine the prognosis of LUAD patients and to find suitable patients for mRNA vaccines.

## Discussion

Lung cancer is the leading cause of cancer deaths^21^, which has a 4–17% 5-year survival rate^22^, and its most common pathological subtype is LUAD. To date, surgical treatment is the primary method for LUAD in its early stages; however, in asymptomatic patients, many surgical opportunities are lost^23,24^. Other treatment options are therefore needed for lung cancer patients who cannot have surgery. Recently, personalized and precise treatments have made notable strides, while other important therapeutic approaches, such as targeted therapy (for example, focusing on EGFR mutations) and immunotherapy, may be employed in similar circumstances^25,26^. However, overcoming EGFR-TKI resistance or poor immune checkpoint expression^27,28^ remains difficult. Therefore, a new approach is needed for precise treatment for LUAD.

Researchers globally have recently paid close attention to the creation of vaccinations for cancer treatment^29^. Considering that malignant cells express both specific and non-specific antigens, the mRNA cancer vaccines are an innovative immunotherapy that shows potential for the treatment of cancer. Patients and preclinical models might both be used as targets for the mRNA vaccination^30–32^. Combining tumor vaccination with ICPs or chemotherapeutic drugs increases therapeutic efficacy^33^, and as a result, increasing numbers of molecular subtypes are being researched to develop cancer therapies. Dixon et al. discovered that ferroptosis, a unique cell death mode different from necrosis and apoptosis, plays an important function in cancer progression^34,35^. Iron is an important participant in various biological processes within cells. Studying the regulation and role of ferroptosis in lung cancer cells can deepen our understanding of the development mechanism of lung cancer, provide important biological markers and prognostic factors, and help to find new treatment strategies and targeted drugs, improve the accuracy of lung cancer diagnosis and treatment effect. However, whether the LUAD antigen is suitable for mRNA cancer vaccines and the correlation between ferroptosis-associated genes and the prognosis of LUAD patients remains unclear. The ferroptosis score—a potential biomarker—may have a considerable impact on molecular subtypes, TME cell-infiltration features, immunotherapy efficacy and the prognosis of LUAD patients^36^.

The FRG profiles and somatic mutations of LUAD were created here, and the results show a variety of potential antigen targets that might account for LUAD. To verify the therapeutic effect of the selected antigens, prognostic roles and ferroptosis connections were further examined. This is due to the possibility that the antigens discovered by utilizing the gene alteration profile are not functionally important in LUAD. Using narrow-down analysis, six tumor antigens (AGPS, NRAS, MTDH, PANX1, NOX4, and PPARD) were found to be related to better prognoses as well as the infiltration of antigen-presenting cells in LUAD. The expression of these genes shows different trends in different TNM stages and an increasing tendency at advanced stages. The results indicate that the recognized tumor antigens might be presented and processed to T cells directly and then recognized by B cells to trigger an immune response. Additionally, these genes may induce ferroptosis in tumor cells to kill more tumor cells, making them promising candidates for an mRNA vaccine.

Since a portion of patients receiving tumor vaccine-based therapy benefit from it and survive^37,38^, patients with LUAD were divided into groups according to their tumor ferroptosis-related gene profiles to determine the best course of action for tumor vaccine-based therapy. Genes that are aberrantly expressed in LUAD were examined to find possible LUAD mRNA vaccines, whereby we found 734 genes that are often mutated and up regulated and might act as tumor antigens. The possibility of the ferroptosis gene acting as an mRNA antigen was then considered. After analysis of OS and PFS, 24 FRGs were identified from 734 potential antigens. Considering the interaction between tumor antigens and APCs, these potential antigens were further screened through the TIMER database where it was found that AGPS, NRAS, MTDH, PANX1, NOX4, and PPARD genes were positively correlated with different APCs. Moreover, the higher expression of these genes had a worse prognosis.

To determine which patients could benefit from vaccination, ferroptosis subtypes could be utilized to reflect the ferroptosis status in the tumor microenvironment. Differential gene expression profiles from TCGA-LUAD and GSE11969 cohorts and clinical prognosis characterized the three ferroptosis subtypes FS1–FS3. We observed that FS1 was associated with a better prognosis, whereas FS2 and FS3 had a lower probability of survival, and FS2 and FS3 subtypes accounted for a larger portion of patients in the middle and late stages of life. Therefore, we infer that the ferroptosis classification could be utilized to estimate the prognosis for LUAD patients and that it is more accurate than that of traditional staging and classification, which has been verified in different cohorts.

Recent analyses have indicated that increased TMB and somatic mutation ratios are associated with enhanced anticancer immunity^39,40^. The TMB, MSI, and total number of mutations were estimated for each LUAD patient by utilizing the mutation dataset of TCGA-LUAD, with FS3 having the highest TMB and total number of mutations, followed by FS2 and FS1. Additionally, 30 genes, including TP53, TTN, MUC16, etc., showed a different mutation status in different subtypes. These results suggest that different ferroptosis subtypes have different mutation features and that TMB and the number of mutations may be used as possible markers for the use of mRNA vaccines.

ICPs, such as PD-L1 and TIM-3^41,42^, and ICD modulators, such as CALR and HMGB1^43–45^, significantly regulate host anti-tumor immunity, influencing the effectiveness of mRNA vaccinations, showing that there is an important relationship between ferroptosis and immune regulation. Additionally, the expressions of ICPs and ICD modulators varied according to the ferroptosis subtype. Similar expression trends of differentially expressed ICD genes in different subtypes were observed; i.e. they were most expressed in FS3, followed by FS2 and FS1. Among these, CXCL10 and HMGB1 showed significant differences between the TCGA-LUAD and GSE11969 cohorts. CXCL10 has significant immunomodulatory effects, such as stimulating monocyte, natural killer cell, and T cell migration and regulating adhesion molecule expression^46^. HMGB1 participates in several biological activities including inflammation, cell differentiation, and tumor cell migration^47^. Moreover, the trend of ICP gene expression was similar in the two cohorts; however, different trends were observed in different ferroptosis subtypes. In conclusion, ICPs could be exploited as biomarkers for mRNA vaccines and ferroptosis type may indicate the expression levels of ICD modulators. Patients with high ICP gene expression may react less effectively to mRNA vaccinations and those with upregulated ICD modulators may react more effectively.

The immunological state of a tumor affects how the body reacts to mRNA vaccinations. By using ssGSEA to score 28 signature genes previously reported in the TCGA-LUAD and GSE11969 cohorts, immune cells components in the three ferroptosis subtypes were identified. The composition of immune cells differed significantly between these subtypes. The scores of activated B cells, macrophages, mast cells, and natural killer cells in FS1 were significantly higher than those in FS2 and FS3, while the scores of activated CD4 T cells and memory B cells in FS3 were higher than those in FS1. Based on the ESTIMATE algorithm, we found that there was more immune and stromal cell infiltration in patients with FS1 type tumors, followed by FS2 and FS3. FS1 has a hot immune phenotype, while FS3 has a cold immune phenotype. The ferroptosis gene expression profile of each patient was also used to construct a LUAD ferroptosis profile to accurately identify the ferroptosis status. Additionally, a WGCNA of ferroptosis related genes was constructed to find the gene enriched in ferroptosis-related functions and pathways, beneficial to the personalized treatment selection of mRNA vaccines.

Our research is the first to identify ferroptosis biomarkers and ferroptosis subtypes in LUAD. The relationship between LUAD-related mutations and its ferroptosis subtypes was also examined. Given that the Food and Drug Administration (FDA) recently approved mRNA vaccines loaded with lipid nanoparticles (LNPs) for the prevention of COVID-19^48^, we anticipate that mRNA vaccines for cancer immunotherapy will advance rapidly.

Nevertheless, the vaccine antigens associated with ferroptosis in LUAD, and other prognostic factors discovered here, should be confirmed by in-vivo research in the future. Additionally, the appropriate mRNA structure modifications, route of administration of mRNA vaccines, its instability, innate immunogenicity, and in-vivo delivery inefficiencies need further research.

## Conclusions

Promising LUAD antigens for the creation of an mRNA vaccine include NRAS, MTDH, PANX1, NOX4, and PPARD. Vaccination is more appropriate for patients with ferroptosis subtype 3. This study provides a theoretical basis for the development of mRNA vaccines for LUAD, the selection of patients to be vaccinated, and the prognosis of these patients.

## Data Availability

All data generated and described in this article are available from the corresponding web servers, and are freely available to any scientist wishing to use them for noncommercial purposes, without breaching participant confidentiality. Further information is available from the corresponding author on reasonable request.

## Abbreviations

LUAD: Lung adenocarcinoma
NSCLC: Non-small cell lung cancer
TCGA: The cancer genome atlas
GEO: Gene expression omnibus
APC: Antigen presenting cell
ICI: Immune checkpoint inhibitor
GDC: Genomic Data Commons
TPM: Transcripts per million
FRG: Ferroptosis-related gene
ICD: Immune cell death
ICP: Immune checkpoint
GEPIA: Gene Expression Profiling Interactive Analysis
TIMER: Tumor immune estimation resource
WGCNA: Weighted correlation network analysis
GSVA: Gene set variation analysis
GSEA: Gene set enrichment analysis
ssGSEA: Single-sample GSEA
PFS: Progression-free survival
OS: Overall survival
TIIC: Tumor immune infiltrating cell
TP53: Tumor protein p53
FS: Ferroptosis subtype
LNP: Lipid nanoparticle
EGFR: Epidermal growth factor receptor
TKI: Tyrosine kinase inhibitor
PD-L1: Programmed cell death 1 ligand 1
CALR: Calreticulin
HMGB1: High mobility group box 1
CXCL10: C-X-C motif chemokine ligand 10
